# Factors Driving Microbial Community Dynamics and Potential Health Effects of Bacterial Pathogen on Landscape Lakes with Reclaimed Water Replenishment in Beijing, PR China

**DOI:** 10.3390/ijerph19095127

**Published:** 2022-04-22

**Authors:** Junzhi Zhang, Xiao He, Huixin Zhang, Yu Liao, Qi Wang, Luwei Li, Jianwei Yu

**Affiliations:** 1 Beijing Climate Change Response Research and Education Center, Beijing University of Civil Engineering and Architecture, Beijing 100044, China; hexiao0365@126.com (X.H.); zhanghuixin_bucea@163.com (H.Z.); yuliao_1994@126.com (Y.L.); li_hany@163.com (L.L.); 2 Key Laboratory of Drinking Water Science and Technology, Research Center for Eco-Environmental Sciences, Chinese Academy of Sciences, Beijing 100085, China; qiwang_st@rcees.ac.cn; 3 University of Chinese Academy of Sciences, Beijing 100019, China

**Keywords:** reclaimed water, health effects, microbial community dynamics, diversity and richness, driven factors, pathogens, networks

## Abstract

Assessing the bacteria pathogens in the lakes with reclaimed water as major influents are important for public health. This study investigated microbial communities of five landscape lakes replenished by reclaimed water, then analyzed driven factors and identified health effects of bacterial pathogens. 16S rRNA gene sequence analysis demonstrated that *Proteobacteria*, *Actinobacteria*, *Cyanobacteria*, *Firmicutes,* and *Verrucomicrobia* were the most dominant phyla in five landscape lakes. The microbial community diversities were higher in June and July than that in other months. Temperature, total nitrogen and phosphorus were the main drivers of the dominant microbial from the Redundancy analysis (RDA) results. Various potential bacterial pathogens were identified, including *Pseudomonas*, *GKS98_freshwater_group*, *Sporosarcina*, *Pseudochrobactrum*, *Streptomyces* and *Bacillus*, etc, some of which are easily infectious to human. The microbial network analysis showed that some potential pathogens were nodes that had significant health effects. The work provides a basis for understanding the microbial community dynamics and safety issues for health effects in landscape lakes replenished by reclaimed water.

## 1. Introduction

Reclaimed water is a special water source has received concentrated concerns in many countries and regions [1,2,3]. Replenishing landscape lakes with reclaimed water is already one of the effective ways to improve water utilization [4]. Over a quarter of the reclaimed water was used to replenish landscape lakes [3,5,6]. However, there still have some obvious differences in the water quality of reclaimed water as compared to natural lakes [4]. Especially, high levels of N, P, disinfection by-products and bacterial pathogens could still remain in reclaimed waters after extensive wastewater treatment, which would cause health issues of water safety easily [6,7,8,9,10]. Thus, the pathogens cause potential threats on the public health, especially to the visitors exposed to these waters such as swimmers or fishers [11,12,13].

Recently, the impact of reclaimed water as a water source has attracted much attention [14]. For the landscape lakes receiving reclaimed water, the water quality and biological community are largely changed. Although the driving factors for microorganism variation in different water environments has been studied [14,15], little is known regarding microorganism dynamics in surface waters with reclaimed water influents. Microbial communities reflect the structure and function of water ecosystems, which are strongly correlated with environmental factors. For example, temperature, pH, light, total nitrogen (TN), and total phosphorus (TP) affect the microbial community greatly [16,17]. Moreover, algal blooms and reproduction may also affect microbial community through nutrients consumptions [18]. Meanwhile, for the reclaimed water, great attention has been paid to the hazard induced by bacterial pathogens [19,20,21]. A variety of bacterial pathogens have been identified and hardly removed by wastewater treatment [22,23], such as *Aeromonas*, *Pseudomonas*, *Mycobacterium*, *Legionella* and pathogenic *E. coli* [13,14]. Thus, together with chemical contaminants, the health effects of pathogens should be considered in the surface waters receiving reclaimed water [4,24,25]. The status of bacterial pathogens during treatment processes have been evaluated in previous studies [9,26,27]. For landscape lakes, it is unexplored that how reclaimed water influences microbial community structure, dynamics of bacterial pathogens.

In this study, the microbial community structure was explored with 16S rRNA gene sequencing for five landscape lakes replenished by reclaimed water. Environmental factor association analysis was employed to identify the environmental driven factors related to the dynamics of bacterial pathogens, which were visualized by redundancy analysis (RDA), Heatmap and microbial network. This study would contribute to the comprehensive evaluation on the hazards of pathogens in more receiving waters due reclaimed water replenishment, and the development of regulation strategy microbial community in reclaimed water.

## 2. Materials and Methods

### 2.1. Water Samples

**Figure 1:**
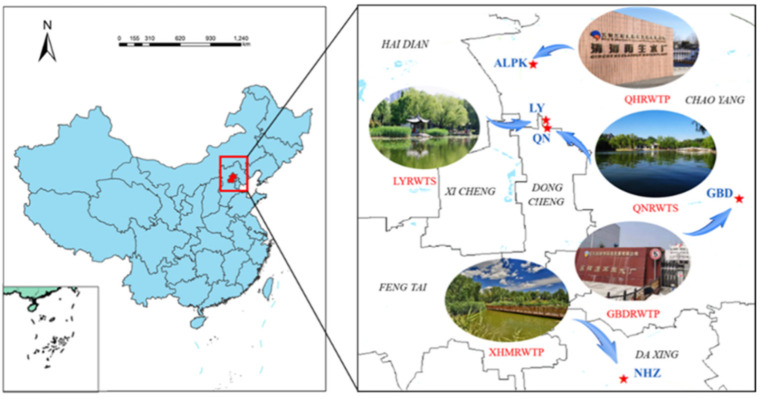
Five sampled landscape lakes in Beijing.

The information about the five investigated landscapes (GBD, ALPK, NHZ, QN and LY in Beijing) are provided in Figure 1. GBD is located in the Tonghui River with an area of 142,900 m^2^ and is replenished by the reclaimed wastewater treatment plant of GBD (GBDRWTP). NHZ as a wetland park was also chosen (300,000 m^2^), which is mainly supplied by the XHM reclaimed wastewater treatment plant (XHMRWTP). ALPK is located on the Dragon River system of Olympic Forest Park with an area of 635,000 m^2^, which is mainly replenished by the effluent of QH reclaimed wastewater treatment plant of (QHRWTP). QN and LY lakes are replenished by the reclaimed wastewater treatment stations of QNRWTS and LYRWTS, which are 611,000 m^2^ and 700,000 m^2^, respectively.

The water depth of the five lakes ranges from 0.5 to 2.5 m. The sampling was carried out once a month from May to September (on the same day for all five lakes), and the parallel samples from the surface and the bottom were collected at the same time. All 50 samples were stored in brown bottles (4 °C in the dark) before subsequent analysis.

### 2.2. Water Quality Analysis

All samples were analyzed as soon as possible within three days. Temperature (T) and pH were recorded simultaneously along with the sampling process. Other water quality parameters were determined according to the EPA standard methods including the total phosphorus (TP), total nitrogen (TN), orthophosphate (PO_4_^3−^), ammonium (NH_4_^+^), and nitrate (NO_3_^−^) [28]. Optical density 680 (OD_680_) was employed to characterize algal cell density by a UV-vis spectrophotometer (Lambda 650s, PerkinElmer, Waltham, MA, USA) at 680 nm. Water quality characteristics are shown in Table 1, the temperature of the five lakes ranged from 23 °C to 30 °C and pH from 7.21 to 8.85, while average values with errors are included for other indicators.

### 2.3. DNA Extraction and High-Throughput Sequencing

The method followed the procedure described in a previous work [29]. The E.Z.N.A.^®^ Water DNA Kit (Omega Bio Tek, Norcross, GA, USA) was used to extract DNA from the samples. The V4-V5 region of the bacterial 16S ribosomal RNA genes was amplified by PCR reactions. The final extension phase was conducted at 72 °C and kept for 10 min until the PCR thermal cycler stopped at 10 °C [30]. PCR reactions were performed in triplicate using a combination of the three PCR products detected by 2% agarose gel electrophoresis. The amplified products were purified and quantified by the AxyPrep DNA Gel Extraction Kit (Axygen Biosciences, Union City, CA, USA) and QuantiFluor™-ST (Promega, Madison, WI, USA) according to the manufacturer’s instructions, respectively.

Purified amplicons were pooled in equimolar amounts and paired-end sequencing (2 × 250) was performed on the Illumina MiSeq platform according to the standard protocol of Majorbio Bio-Pharm Technology Co., Ltd., Shanghai, China. The original reads of Accession Number: PRJNA759710 were readily available in the database NCBI Sequence Read Archive (SRA).

### 2.4. Data Processing and Statistical Analysis

The index sequence was used to distinguish the data for each sample and the QIIME2 (version 4.1) was employed to analyze the data. Sequencing was completed by the fastp software and FLASH software. The sequence de-hybridization method and parameters are shown in SI (Section S1).

The maximum mismatch rate allowed for overlapping regions of spliced sequences was 0.2 to screen out inconsistent sequences. Sequences with 97% similarity to any other sequence were classified as an operant classification unit (OTU) using UPARSE (version 7.1) software. Classification analysis of each 16S rRNA gene sequence was performed in the SILVA (SSU118) 16S rRNA database using a 70% confidence threshold.

Since the five lakes are all shallow and their stratification differences were insignificant, the data from 0.5 m and 2.5 m depth were averaged for analysis. OTU data was used for the statistical analysis of sequencing results. some rare taxa were removed to normalize the data. The microbial communities of landscapes lakes as well as their driving factors were analyzed in multiple ways. The richness (Sobs) and diversity (Shannon) indexes were calculated by the MOTHUR package (version 1.30.1), for describing the alpha diversity of the microbial communities. More environmentally relevant factors were selected using Variance Inflation Factors (VIFs), calculated as VIF_i_ = 1/(1 − R_i_^2^). Where R_i_^2^ represents the proportion of variance of the ith independent variable in the model that is correlated with the other independent variables. R_i_ was used to determine the covariance between the ith independent variable and the other independent variables. RDA and Spearman correlation Heatmap analysis was performed using pheatmap package based on R software to visualize the relationship between dominant community composition and environmental factors. BugBase was used to analyze the abundance of potential pathogens. The microbial network correlations were analyzed by microbial network analysis. A Correlation Network Figure was determined based on graph theory knowledge to analyze the correlation of the biological network using Networkx.

## 3. Results and Discussion

### 3.1. Microbial Community of Examined Lakes

A total of 52 classes belonging to 27 distinct phyla were recognized in the samples. Figure S2 gives the relative abundance of the dominant genera, indicating that the variation in relative abundance among the dominant genera varied considerably. The phylum of *Proteobacteria* was the most abundant phylum in the examined lakes, accounting for 41.78% of all the sample sequences. *Cyanobacteria* was the second most abundant phylum, accounted for 17.21% of the total phyla. The rest of the sequences belonged to *Firmicutes* (12.49%), *Actinobacteria* (12.31%), *Verrucomicrobia* (6.71%), *Bacteroidetes* (3.88%), *Planctomycetes* (3.11%) and others (2.51%, <1% for each). *Proteobacteria* has been identified as the dominant phylum either in natural water or reclaimed water, while the abundance of *Firmicutes* in reclaimed waters was higher than in natural waters [29,31,32,33]. Therefore, *Firmicutes* in the landscape lakes replenished by reclaimed water was one of the four dominant phyla (>10%), over the abundance of *Actinobacteria* [34,35].

### 3.2. Microbial Community Diversity and Potential Bacterial Pathogens

**Figure 2:**
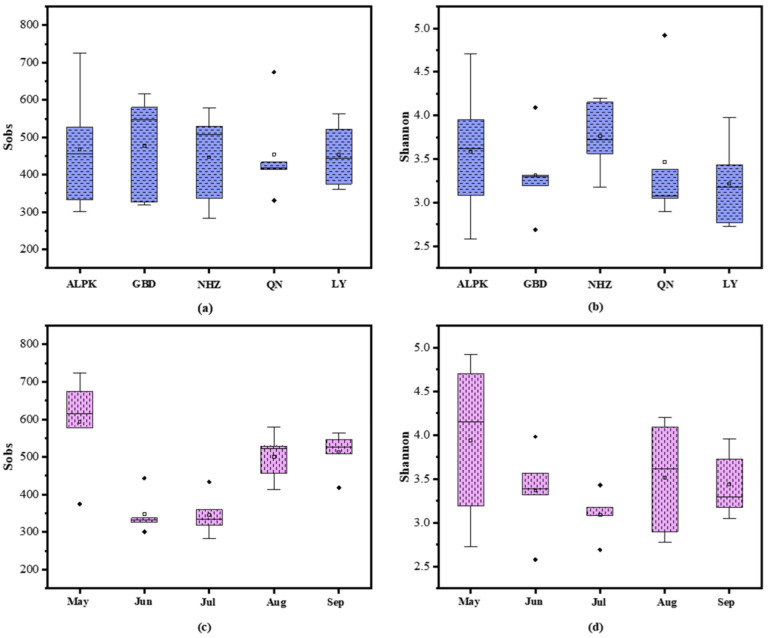
OTU richness (Sobs) and diversity (Shannon) of all samples in different locations (**a**,**b**) and different months (**c**,**d**).

The microbiome survey yielded 2, 695, 332 reads from 50 samples. After normalization, a total of 1114 OTUs were obtained and verified by rarefaction curves. The end of the rarefaction curves gradually flattened out, suggesting that the detection rate of microbial communities was reaching saturation in all samples. The standardized Sobs index and Shannon index from normalized OTUs results were used to assess the richness (Figure 2a,c) and diversity (Figure 2b,d).

The microbial richness and diversity were not considerably varied across examined sample sites but diverged between months. From Figure 2a,b, the microbial Sob indexes were between 447 to 477 averagely and the Shannon indexes were between 3.22 to 3.76 averagely in different sites. As shown in Figure 2c,d, the Sobs indexes were 347 and Shannon indexes were 3.23 on average in June and July (28 °C~31 °C), while Sobs and Shannon indexes were 535.5 and 3.63 in May, August and September (20 °C~25 °C). The Sobs indexes in May, August and September were 1.54 times of June and July The diversities were higher and the richness was lower of microbial communities in June and July than them in other months. The index of similarities was calculated using ANOSIM/Adonis method (Figure S2), and the reliability was good (*p* values < 0.05). From the corresponding parameters listed in Table S2, it could be seen that sampling time (in month) (R^2^ = 0.27) had a greater effect on microbial community composition than location (R^2^ = 0.16).

**Figure 3:**
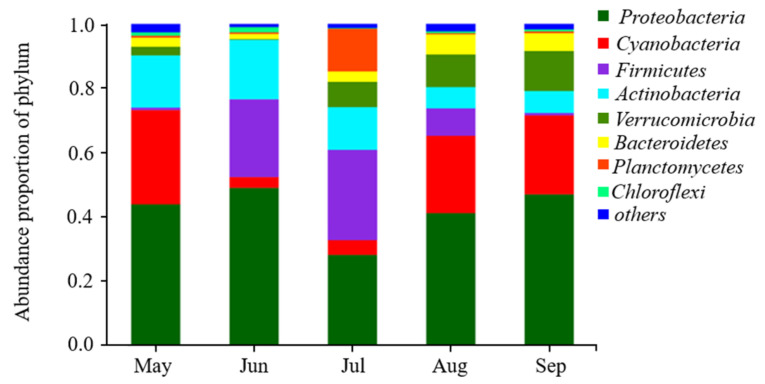
Taxonomic of microbial community in different months.

The taxonomic of the microbial community in each month is shown in Figure 3. It could be seen that the proportion of *Firmicutes* was much higher than that in May, August and September In July, the proportion of *Firmicutes* was 28%, which equal to that of *Proteobacteria* and is the most abundant phylum. As shown in Figure 3, *Proteobacteria* and *Actinobacteria* were significantly reduced from June to July In previous studies, *Firmicutes* genera has been found to be capable of tolerating and thriving at high temperature [36]. During algal blooms in June and July, *Firmicutes* were better adapted to the dissolved oxygen depletion in water than *Proteobacteria* and *Actinobacteria*. Meanwhile, from Table 1, TN of the five investigated landscape lakes in this study was several times higher than that of the surface water quality standard, which might be due to the replenishment of reclaimed water [37,38]. High N concentrations has been found to be suitable for the growth and propagation of some *Firmicutes* genera, which could be a reason for the increase in abundance of *Firmicutes*. Additionally, as *Firmicutes*, contains several pathogens such as *Staphylococcus* and *Bacillus*, special attention should be paid for its abundance in waters. These pathogens induce health hazards through parasitizing the skin, nasal cavity, throat, gut, canker sores, abscesses. In the examined landscape lakes, the increased abundance of *Firmicutes* in June and July could elevate the hazards of diseases such as wound infections and diarrhea in humans [26,27,39].

Compared to *Firmicutes*, *Proteobacteria* and *Actinobacteria* were commonly frequent phyla. *Proteobacteria* was the most dominant phylum each month (over 40% averagely). *Actinobacteria* was a common phylum and the average abundance in each month was over 10%. *Proteobacteria* and *Actinobacteria* have been widely detected in natural water, and could constitute more than half of the total bacteria in some surface waters, while the results of this study showed that the proportion of *Proteobacteria* and *Actinobacteria* in examined lakes was lower than in natural water. As the microbial composition is closely related to environmental factors, thus reclaimed water replenishing could affecting microbial community by changing environmental factors. Therefore, only further studies on the relationship between environmental factors and microbial community could better predict the dynamics. Moreover, the study found that bacteria such as *Legionella* exist in the *Proteobacteria* while *Rhodococcus* present in *Actinobacteria*, which have been recognized as emerging pathogens in reclaimed water sources [40,41,42]. Studies for health effects also found that these pathogens could lead to lung disease [43,44].

### 3.3. Responses of Community Changes and Potential Bacterial Pathogens to Key Environmental Factors

**Figure 4:**
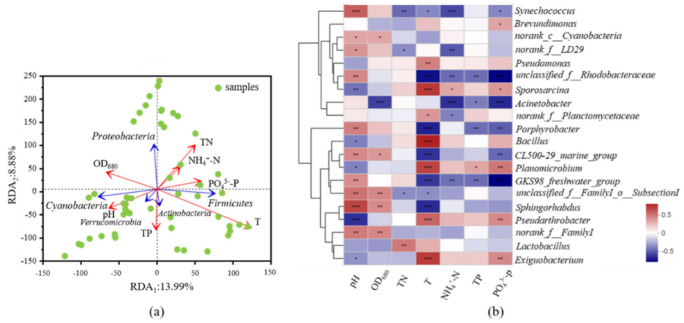
RDA analysis of environmental factors and the microbial community (**a**); Heatmap analysis of key environmental factors and microbial community with top 20 genera (**b**), * 0.01 < *p* ≤ 0.05, ** 0.001 < *p* ≤ 0.01, *** *p* ≤ 0.001.

A vector plot of environmental variables correlated to the changes in microbial community (calculate using RDA analysis) is shown in Figure 4a, and the Spearman correlation Heatmap of environmental factors and major genera were shown in Figure 4b. To improve the accuracy of RDA analysis, several critical environmental factors were chosen based on VIF analysis (VIF < 10) [45], and Heatmap analysis was conducted on the genera to explore the microbial community and bacteria pathogen changes (Figure 4b).

As shown in Figure 4a, RDA analysis showed that T, TN, TP, NH_4_^+^-N and PO_4_^3−^-P had considerable influences on the dominant phyla. T (*p* = 0.001) and TN (*p* = 0.097) were the two dominant driven factors affecting the dominant phyla. T was positively correlated with *Firmicutes*, and negatively correlated with *Proteobacteria*, which was aligned with the changes of the dominant phyla with month variations in Figure 3. It could be seen that the *Proteobacteria* were negatively correlated with T (Figure 4a), and its abundance in July was significantly decreased (Figure 3), which could be attributed to the that *Firmicutes* became the dominant phylum and occupying the survival of *Proteobacteria*. Previous studies showed that some genera of *Firmicutes* have a strong ability to tolerate high temperatures [46], which could be the reason for the dominance of *Firmicutes* in June and July Nitrogen was positively correlated with *Firmicutes*. *Firmicutes,* as nitrogen-active strains, can adapt to higher nitrogen levels due to the influent of reclaimed water [46]. Compared to temperature, the nitrogen effect on *Firmicutes* was minor. This phenomenon could be associated to that nitrogen was the limiting factor for *Firmicutes* [34,35]. In Figure 4a, the temperature was positively correlated with *Actinobacteria,* and nitrogen was negatively correlated, indicating that *Actinobacteria* cannot well adapted to landscape lakes with high nitrogen levels. Similarly, the abundance of *Actinobacteria* in this study was lower than that in natural water [31,34,35].

Heatmap analysis was conducted on genera to further explore the microbial community structure. The top 20 genera were selected and analyzed with Heatmap in (Figure 4b). The important genera of *Firmicutes* were *Bacillus*, *Sporosarcina*, *Lactobacterium*, *Exiguobacterium,* and *Planomicrobium*, all of them were positively correlated with T. Several genera in *Proteobacteria* were negatively or poorly correlated with T and significantly negatively correlated with PO_4_^3−^-P. For *Proteobacteria*, PO_4_^3−^-P is usually a limiting factor [47,48]. In addition, several genera containing human pathogens were discovered, such as *Bacillus*, *Acinetobacter,* and *Pseudomonas*. Therefore, the analysis of the top 50 genera in relation to environmental factors and the identification of bacterial pathogens among them was continued (Figure S1). Many bacteria genera (*Acinetobacter*, *Bacillus*, *Pseudomonas*, *Mycobacterium*, *Escherichia-Shigella*, *Rhodococcus* and *Legionella*) have been found potential pathogenic. *Pseudomonas* are widely distributed in soil, water, food and air, including several bacterial pathogens such as *P. fluorescens* [49,50], which could cause wounds infections [50,51]. Since *Pseudomonas* was the dominant genus in landscape lakes replenished by reclaimed water, its potential hazard should be further noticed. Furthermore, *Mycobacterium*, *Rhodococcus* and *Legionella* are all lung pathogens that caused lung and respiratory infections [13,26,27,52]. Additionally, *Rhodococcus* and *Legionella* were positively correlated with T and PO_4_^3−^-P. As reclaimed waters usually have higher P level than natural waters, thus potential safety issues should be paid close attention. Previous studies showed that TN in landscape lakes influenced by the reclaimed water was usually high, which could make *Mycobacterium*, *Pseudomonas*, *Legionella sp.* and *Streptococcus* more active, especially during high temperature month when algal blooms were more possible to occur. Higher activity of bacterial pathogens increases the hazard of disease in humans. In addition, with similar levels of TN and other nutrients, *Mycobacterium*, *Pesudomonas*, *Legionella* and *Brevundimonas* have found to be dominantd in lakes with reclaimed water impacts, and these pathogens could attach to suspended particles to increase their pathogenic hazards [53,54].

### 3.4. Health Effects of Potential Pathogens and Microbial Co-Occurrence

**Figure 5:**
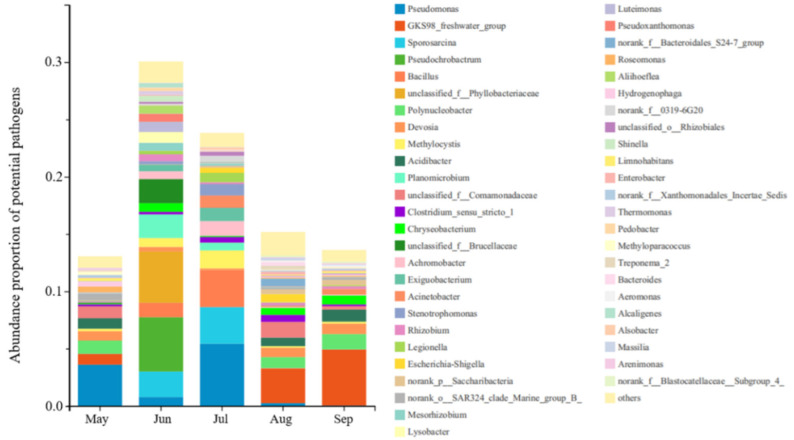
Abundance proportion of potential pathogens on genus level.

The abundance of potential pathogens in the top 50 genera was analyzed using the BugBase in functional prediction. Figure 5 shows that the species and the abundance of bacteria pathogens were higher in June and July than that in the other three months. The abundance of potential pathogens in June and July was about 30% of total genera, while that in May, August and September were about 15%. The top five genera that contributed to potential pathogenicity were: *Pseudomonas*, *GKS98_freshwater_group*, *Sporosarcina*, *Pseudochrobactrum* and *Bacillus*. *GKS98_freshwater_group* (*Proteobacteria*) was the high contribution pathogenic genus in August and September *Pseudomonas* (*Proteobacteria*), *Pseudochrobactrum* (*Proteobacteria*) and *Sporosarcina* (*Firmicutes*) were the main pathogenic genera in June and July This result indicate that the microbial community composition was significant varied, which could be attributed to environmental factors changes in June and July In addition, several species of pathogens (*Kocuria rosea*, *Brevundimonas diminuta* and *Chryseobacteriun indologenes*) have been found in May to July [55]. *Kocuria rosea* colonizes oropharynx, skin and mucous membrane leading to endocarditis [56,57]. *Chryseobacterium indologenes* is an uncommon human pathogen, which causes severe infections such as septicaemia and ventilator-associated pneumonia in immunocompromised patients [58,59]. *Kocuria rosea* (*p* = 0.00015) and *Brevundimonas diminuta* (*p* = 0.00076) were positively correlated with T and *Chryseobacteriun indologenes* was also positively correlated with TN (*p* = 0.0003).

**Figure 6:**
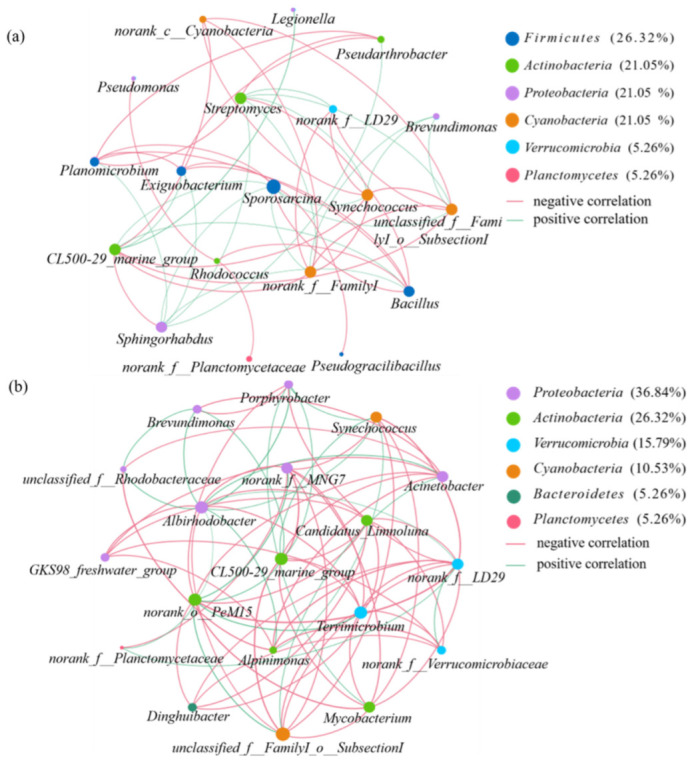
Co-occurrence networks for microbial communities in May June and July (**a**); in August and September (**b**).

In addition to environmental factors, microbial co-occurrence patterns were an important driver shaping community. The main pathogenic contributing genera in May to July were *Pseudomonas*, *Sporosarcina* and *Bacillus* while those in August to September were *GKS98_freshwater_group* (Figure 5). Accordingly, microbial co-occurrence networks were constructed in May to July (Figure 6a) and from August to September (Figure 6b), respectively. There were 19 nodes with *p* < 0.05 in the group of May to July, 18 nodes with *p* < 0.05 in the group of August to September *p* < 0.05, suggesting that the nodes have relevance in the microbial network and they were necessary for the network. Most of the nodes in both groups belonged to *Proteobacteria, Firmicutes*, *Actinobacteria* and *Cyanobacteria*, consistent with the microbial community results. According to the Heatmap results, most of the important nodes were strongly correlated with T, TN and TP, which indicated that environmental factors could affect microbial networks through nodes. Focusing on the microbial network influenced by these nodes, it should be highlight that many of them were pathogenic [60,61]. As shown in Figure 6, potential genera pathogens with high abundance from May to July of microbial networks included *Sporosarcina*, *Bacillus*, *Pseudomonas*, *Streptomyces*, *Legionella* and *Rhodococcus*. In addition, from August to September included *GKS98_freshwater_group*, *Acinetobacter* and *Mycobacterium*.

More than highlighting important nodes microbial network also showed the co-occurrence relationship between the nodes. In Figure 6, both microbial networks had more promoting links relationships than inhibiting links. Genera belonging to the same phylum were mostly in a promoting relationship with each other, which was one reason why the rapid increase of a particular phylum in a month. However, some genera pathogens were also mostly promoting relationships with genera belonging to the same phylum (whether pathogenic or not), such as *Bacillus*, *Pseudomonas*, *Rhodococcus* and *Mycobacterium*. When environmental factors induce changes in the abundance of microbial communities, it is also an opportunity for bacteria pathogens to multiply. The higher abundance of nodal genera, the higher hazard for health effects due to genera pathogens would be posed. In terms of the number of links, there were much more connections in the second network (Figure 6b from August to September) than in the first network (Figure 6a from May to July), and the increased density of connections in the community made it a more complex network. Complex networks mean more stable relationships [31,62]. In a simple network, the structure changes are hard to predict, and the pathogenic hazard is higher [31,62]. Environmental factors were inextricably linked to microbial networks, and result of the current work highlights the importance of studying microbial networks based on environmental factors.

## 4. Conclusions

Microbial community composition of five landscape lakes replenished by reclaimed water were investigated. The conclusions are described as follows: the microbial community composition and diversity in the landscape lakes fluctuated in different months. *Proteobacteria*, *Cyanobacteria*, *Firmicutes* and *Actinobacteria* were the most dominant phyla, the diversity and richness of which were associated with time variations. The key environmental factors affecting the microbial community were temperature, nitrogen and phosphorus, and the temperature was the most significant. Many genera were found to be pathogenic, including *Acinetobacter*, *Mycobacterium*, *Pseudomonas*, *Rhodococcus* and *Legionella*, of which, *Kocuria rosea*, *Brevundimonas diminuta* and *Chryseobacteriun indologenes* were identified. Meanwhile, the network analysis indicated that genera pathogens already have a certain position in the network, indicating an increased potential hazard of pathogenicity in the microbial networks. Therefore, for the landscape lakes with reclaimed water influent, the potential health hazards induced by reclaimed water should be extensively assessed. The seasonal changes in microbial communities during May to September were identified here, however, the variation of microbial communities in the rest of the seasons still missing. In addition, the rRNA sequencing has low precision in identifying species-level microbial, making the available data are still scarce. Therefore, future studies may design a sampling throughout the whole year and some methodological updates and more highly accurate analysis tools should be considered.

## Figures and Tables

**Table 1:** Water quality characteristics.

Parameters	Value	Parameters	Value
T (°C)	23.0~30.0	TN (mg/L)	3.70 ± 0.87
pH	7.21~8.85	TP (mg/L)	0.024 ± 0.009
NH_4_^+^-N (mg/L)	0.0851 ± 0.0012	PO_4_^3−^-P (mg/L)	0.0177 ± 0.0003
NO_3_^−^ (mg/L)	5.31 ± 0.55	OD_680_ (cm^−1^)	0.457 ± 0.00

## Data Availability

All data and materials support their published claims and comply with field standards. The original reads of Accession Number: PRJNA759710 were readily available in the database NCBI Sequence Read Archive (SRA).

## Supplementary Materials

The following supporting information can be downloaded at: https://www.mdpi.com/article/10.3390/ijerph19095127/s1, Figure S1: Pie pilot of microbial community on phylum level. Figure S2: Analysis of similarities of microbial community in different groups on OTU level. The corresponding Adonis analysis results exhibited in Table S2. Figure S3: Spearman correlation Heatmap of primary environmental factors and bacterial community with top 50 bacterial community abundance. Table S1: TN in different lakes and different layers in five months (unit: mg/L). Table S2: Adonis analysis of microbial community in different factor on OTU level.

## References

[1] Chen Z., Tong Y., Ngo H.H., Yun L., Li G.Q., Wu Q.Y., Li K.X., Bai Y., Liu S.M., Hu H.Y. (2018). Assimilable organic carbon (AOC) variation in reclaimed water: Insight on biological stability evaluation and control for sustainable water reuse. Bioresour. Technol..

[2] Ren X.L., Chen H.B. (2021). Effect of residual chlorine on the interaction between bacterial growth and assimilable organic carbon and biodegradable organic carbon in reclaimed water. Sci. Total Environ..

[3] Li D.Q., Huang D., Guo C.F., Guo X.Y. (2014). Multivariate statistical analysis of temporal–spatial variations in water quality of a constructed wetland purification system in a typical park in Beijing, China. Environ. Monit. Assess..

[4] Liu W., Xu Z.Q., Long Y.J., Feng M.Q. (2021). Replenishment of urban landscape ponds with reclaimed water: Spatiotemporal variations of water quality and mechanism of algal inhibition with alum sludge. Sci. Total Environ..

[5] Marks J.S. (2006). Taking the public seriously: The case of potable and non-potable reuse. Desalination.

[6] Zhu L.B., Torres M., Betancourt W.Q., Sharma M., Shirley A., Micallef S.A., Gerba C., Sapkota A.R., Sapkota A., Parveen S. (2019). Incidence of fecal indicator and pathogenic bacteria in reclaimed and return flow waters in Arizona, USA. Environ. Res..

[7] Yue L.Y., Kong W.D., Ji M.K., Liu J.B., Morgan-Kiss R.M. (2019). Community response of microbial primary producers to salinity is primarily driven by nutrients in lakes. Sci. Total Environ..

[8] Malayil L., Ramachandran P., Chattopadhyay S., Cagle R., Hittle L., Ottesen A., Mongodin E.F., Sapkota A.R. (2020). Metabolically-active bacteria in reclaimed water and ponds revealed using bromodeoxyuridine DNA labeling coupled with 16S rRNA and shotgun sequencing. Water Res..

[9] Caicedo C., Rosenwinkel K.H., Exner M., Verstraete W., Suchenwirth R., Hartemann P., Nogueira R. (2019). Legionella occurrence in municipal and industrial wastewater treatment plants and risks of reclaimed wastewater reuse: Review. Water Res..

[10] Kulkarni P., Olson N.D., Paulson J.N., Pop M., Maddox C., Claye E., Rosenberg Goldstein R.E., Sharma M., Gibbs S.G., Mongodin E.F. (2018). Conventional wastewater treatment and reuse site practices modify bacterial community structure but do not eliminate some opportunistic pathogens in reclaimed water. Sci. Total Environ..

[11] Solaiman S., Micallef S.A. (2021). Aeromonas spp. diversity in U.S. mid-Atlantic surface and reclaimed water, seasonal dynamics, virulence gene patterns and attachment to lettuce. Sci. Total Environ..

[12] Pepper I.L., Gerba C.P. (2018). Risk of infection from Legionella associated with spray irrigation of reclaimed water. Water Res..

[13] Hamilton K.A., Hamilton M.T., Johnson W., Jjemba P., Bukhari Z., LeChevallier M., Haas C.N. (2018). Health risks from exposure to Legionella in reclaimed water aerosols: Toilet flushing, spray irrigation, and cooling towers. Water Res..

[14] Banda J.F., Zhang Q., Ma L.Q., Pei L.X., Du Z.R., Hao C.B., Dong H.L. (2021). Both pH and salinity shape the microbial communities of the lakes in Badain Jaran Desert, NW China. Sci. Total Environ..

[15] Aping N., Song L.Y., Xiong Y.H., Lu C.J., Junaid M., Pei D.S. (2019). Impact of water quality on the microbial diversity in the surface water along the three Gorge reservoir (Tgr), China. Ecotoxicol. Environ. Saf..

[16] Zhang Y.T., Shen H., He X.H., Thomas B.W., Lupwayi N.Z., Hao X.Y., Thomas M.C., Shi X.J. (2017). Fertilization shapes bacterial community structure by alteration of soil pH. Front. Microbiol..

[17] Guo Z.B., Liu H., Wan S.X., Hua K.K., Wang D.Z., Guo X.S., He C.L. (2019). Fertilisation practice changes rhizosphere microbial community structure in the agroecosystem. Ann. Appl. Biol..

[18] Yang Y.Y., Chen J.F., Chen X.L., Jiang Q.S., Liu Y., Xie S.G. (2021). Cyanobacterial Bloom Induces Structural and Functional Succession of Microbial Communities in Eutrophic Lake Sediments. Environ. Pollut..

[19] Kevin P., Wang J., Fernando S.C., Thompson J.R. (2014). Secondary metabolite gene expression and interplay of bacterial functions in a tropical freshwater cyanobacterial bloom. ISME J..

[20] Wang K., Razzano M., Mou X.Z. (2020). Cyanobacterial blooms alter the relative importance of neutral and selective processes in assembling freshwater bacterioplankton community. Sci. Total Environ..

[21] Zhang H.H., Jia J.Y., Chen S.N., Huang T.L., Wang Y., Zhao Z.F., Feng J., Hao H.Y., Li S.L., Ma X.X. (2018). Dynamics of Bacterial and Fungal Communities during the Outbreak and Decline of an Algal Bloom in a Drinking Water Reservoir. Int. J. Environ. Res. Public Health.

[22] Gerba C.P., Betancourt W.Q., Kitajima M., Rock C.M. (2018). Reducing uncertainty in estimating virus reduction by advanced water treatment processes. Water Res..

[23] Costán-Longares A., Montemayor M., Payán A., Méndez J., Jofre J., Mujeriego R., Lucena F. (2008). Microbial indicators and pathogens: Removal, relationships and predictive capabilities in water reclamation facilities. Water Res..

[24] Steele M., Odumeru J. (2004). Irrigation Water as Source of Foodborne Pathogens on Fruit and Vegetables. J. Food Prot..

[25] Deviller G., Lundy L., Despo F.K. (2020). Recommendations to derive quality standards for chemical pollutants in reclaimed water intended for reuse in agricultural irrigation. Chemosphere.

[26] Hartemann P., Hautemaniere A. (2011). Legionellosis prevention in France. Bundesgesundheitsblatt.

[27] Nogueira R., Utecht K.U., Exner M., Verstraete W., Rosenwinkel K.H. (2016). Strategies for the reduction of Legionella in biological treatment systems. Water Sci. Technol..

[28] U.S. Environmental Protection Agency (USEPA) (1989). Risk Assessment Guidance for Superfund. Human Health Evaluation Manual, Part A.

[29] Zhang J.Z., Zhang H.X., Li L.W., Wang Q., Yu J.W., Chen Y.S. (2020). Microbial community analysis and correlation with 2-methylisoborneol occurrence in landscape lakes of Beijing. Environ. Res..

[30] Chen Z.C., Zhang X.L., Yang Y., Zhou K., Wragg N., Liu Y., Lewis M., Liu C.Q. (2017). Fabrication and characterization of 3D complex hydroxyapatite scaffolds with hierarchical porosity of different features for optimal bioactive performance. Ceram. Int..

[31] Sadeghi J., Chaganti S.R., Shahraki A.H., Heath D.D. (2021). Microbial community and abiotic effects on aquatic bacterial communities in north temperate lakes. Sci. Total Environ..

[32] Becerra-Castro C., Gonçalo M., Silva A.M.T., Manaia C.M., Nunes O.C. (2016). Proteobacteria become predominant during regrowth after water disinfection. Sci. Total Environ..

[33] Newton R.J., Jones S.E., Eiler A., McMahon K.D., Bertilsson S. (2011). Guide to the Natural History of Freshwater Lake Bacteria. Microbiol. Mol. Biol..

[34] Mondol M.A., Shin S.J., Islam M.T. (2013). Diversity of secondary metabolites from marine bacillus species: Chemistry and biological activity. Mar. Drugs.

[35] Zheng Y., Yang J., Amalfitano S., Yu X.Q., Liu L.M. (2014). Effects of water stratification and mixing on microbial community structure in a subtropical deep reservoir. Sci. Rep..

[36] Woodhouse J.N., Ongley S.E., Brown M.V., Neilan B.A. (2013). Microbial diversity and diazotrophy associated with the freshwater non-heterocyst forming cyanobacterium lyngbya robusta. J. Appl. Phycol..

[37] Sepa (2002). Environmental Quality Standard for Surface Water (Eqssw), Gb3838–2002.

[38] Jing S., Ji D.F., Lin M., Chen Y.Q., Sun Y.Y., Huo L.L., Zhu J.C., Xi B.D. (2017). Developing Surface Water Quality Standards in China. Resour. Conserv. Recycl..

[39] Theodorou C.M., Stokes S.C., Hegazi M.S., Brown E.G., Saadai P. (2020). Is pseudomonas infection associated with worse outcomes in pediatric perforated appendicitis?. J. Pediatr. Surg..

[40] Dong P.Y., Cui Q.J., Fang T.T., Huang Y., Wang H. (2019). Occurrence of antibiotic resistance genes and bacterial pathogens in water and sediment in urban recreational water. J. Environ. Sci..

[41] Cui Q.J., Fang T.T., Huang Y., Dong P.Y., Wang H. (2017). Evaluation of bacterial pathogen diversity, abundance and health risks in urban recreational water by amplicon next-generation sequencing and quantitative PCR. J. Environ. Sci..

[42] Ranjbar M., Behrouz B., Norouzi F., Gargari S.L.M. (2019). Anti-Pcrv Igy antibodies protect against pseudomonas aeruginosa infection in both acute pneumonia and burn wound models. Mol. Immunol..

[43] El-Saed A., Balkhy H.H., Al-Dorzi H.M., Khan R., Rishu A.H., Arabi Y.M. (2013). Acinetobacter is the most common pathogen associated with late-onset and recurrent ventilator-associated pneumonia in an adult intensive care unit in saudi arabia. Int. J. Infect. Dis..

[44] Ebringer A., Rashid T., Wilson C., Shoenfeld Y., Agmon-Levin N., Rose N.R. (2015). Chapter 29—Multiple sclerosis and creutzfeldt-jakob disease are autoimmune diseases probably caused by exposure to the nasal microbe Acinetobacter. Infection and Autoimmunity.

[45] Te S.H., Tan B.F., Thompson J.R., Gin K.Y.H. (2017). Relationship of microbiota and cyanobacterial secondary metabolites in planktothricoides-dominated bloom. Environ. Sci. Technol..

[46] Cuzman O.A., Rescic S., Richter K., Wittig L., Tiano P. (2015). Sporosarcina pasteurii use in extreme alkaline conditions for recycling solid industrial wastes. J. Biotechnol..

[47] Wang H., Liu X., Wang Y., Zhang S., Zhang G., Han Y., Li M., Liu L. (2023). Spatial and temporal dynamics of microbial community composition and factors influencing the surface water and sediments of urban rivers. J. Environ. Sci..

[48] Bunse C., Bertos-Fortis M., Sassenhagen I., Sildever S., Sjöqvist C., Godhe A., Gross S., Kremp A., Lips I., Lundholm N. (2016). Spatio-temporal interdependence of bacteria and phytoplankton during a baltic sea spring bloom. Front. Microbiol..

[49] Bleves S., Viarre V., Salacha R., Michel G.P.F., Filloux A., Voulhoux R. (2010). Protein secretion systems in pseudomonas aeruginosa: A wealth of pathogenic weapons. Int. J. Med. Microbiol..

[50] Curran C.S., Bolig T., Torabi-Parizi P. (2018). Mechanisms and targeted therapies for Pseudomonas aeruginosa lung infection. Am. J. Respir. Crit. Care Med..

[51] Casciaro B., d’Angelo I., Zhang X., Loffredo M.R., Conte G., Cappiello F., Quaglia F., Di Y.P.P., Ungaro F., Mangoni M.L. (2019). Poly (Lactide-Co-Glycolide) Nanoparticles for prolonged therapeutic efficacy of esculentin-1a-derived antimicrobial peptides against pseudomonas aeruginosa lung infection: In vitro and in vivo studies. Biomacromolecules.

[52] Savini V., Fazii P., Favaro M., Astolfi D., Polilli E., Pompilio A., Vannucci M., D’Amario C., Bonaventura G.D., Fontana C. (2012). Tuberculosis-Like pneumonias by the aerobic actinomycetes Rhodococcus, Tsukamurella and Gordonia. Microbes Infect..

[53] Zhang H.H., Wang Y., Chen S.N., Zhao Z.F., Feng J., Zhang Z.H., Lu K.Y., Jia J.Y. (2018). Water Bacterial and Fungal Community Compositions Associated with Urban Lakes, Xi’an, China. Int. J. Environ. Res. Public Health.

[54] Fang T.T., Cui Q.J., Huang Y., Dong P.Y., Wang H., Liu W.-T., Ye Q.H. (2018). Distribution Comparison and Risk Assessment of Free-Floating and Particle-Attached Bacterial Pathogens in Urban Recreational Water: Implications for Water Quality Management. Sci. Total Environ..

[55] Chattopadhyay S., Perkins S.D., Shaw M., Nichols T.L. (2017). Evaluation of exposure to brevundimonas diminuta and Pseudomonas aeruginosa during showering. J. Aerosol. Sci..

[56] Montoya J.E.M., Moran M.A.M., Ardila J.A.B., Henao P.G.C., Rodriguez E.E.M., Meza G.A.C. (2017). Brain abscess by Kocuria Rosea: Case report and literature review. Interdiscip. Neurosurg..

[57] Moreira J.S., Riccetto A.G.L., Silva M.T.N.D., Vilela M.M.D.S. (2015). Endocarditis by Kocuria Rosea in an immunocompetent child. Braz. J. Infect. Dis..

[58] Baruah M., Lyngdoh C., Lyngdoh W.V., Talukdar R. (2016). Noncatheter-related bacteraemia due to Chryseobacterium Indologenes in an immunocompetent patient. Indian J. Med. Microbiol..

[59] Bhuyar G., Jain S., Shah H., Mehta V.K. (2012). Urinary tract infection by Chryseobacterium Indologenes. Indian J. Med. Microbiol..

[60] Hogenhout S.A., Loria R. (2008). Virulence mechanisms of Gram-Positive plant pathogenic bacteria. Curr. Opin. Plant Biol..

[61] Loria R., Kers J., Madhumita J. (2006). Evolution of plant pathogenicity in Streptomyces. Annu. Rev. Phytopathol..

[62] Chen S., Zhang B., Ning D.L., Zhang Y., Dai T.J., Wu L.W., Li T.L., Liu W., Zhou J.Z., Wen X.H. (2021). Seasonal dynamics of the microbial community in two full-scale wastewater treatment plants: Diversity, composition, phylogenetic group based assembly and co-occurrence pattern. Water Res..

