# Supplementary material for: Factors Driving Microbial Community Dynamics and Potential Health Effects of Bacterial Pathogen on Landscape Lakes with Reclaimed Water Replenishment in Beijing, PR China
Source: Int J Environ Res Public Health. 2022 Apr 22;19(9):5127. doi: 10.3390/ijerph19095127 (PMC9106022; doi:10.3390/ijerph19095127)
Supplement: Supplementary file 1 [file ijerph-19-05127-s001.zip › ijerph-1657077-supplementary.pdf]

# Factors Driving Microbial Community Dynamics and Potential Health Effects of Bacterial Pathogen on Landscape Lakes with Reclaimed Water Replenishment in Beijing, PR China

**Junzhi Zhang [1,*], Xiao He [1], Huixin Zhang [1], Yu Liao [1], Qi Wang [2,3], Luwei Li [1] and Jianwei Yu [2,3,*]**

[1] Beijing Climate Change Response Research and Education Center, Beijing University of Civil Engineering and Architecture, Beijing 100044, China; hexiao0365@126.com (X.H.); zhanghuixin_bucea@163.com (H.Z.); yuliao_1994@126.com (Y.L.); li_hany@163.com (L.L.)
[2] Key Laboratory of Drinking Water Science and Technology, Research Center for Eco-Environmental Sciences, Chinese Academy of Sciences, Beijing 100085, China; qiwang_st@rcees.ac.cn
[3] University of Chinese Academy of Sciences, Beijing 100019, China
* Correspondence: zhangjunzhi@bucea.edu.cn (J.Z.); jwyu@rcees.ac.cn (J.Y.); Tel.: +86-010-61209207 (J.Z.); +86-010-62849149 (J.Y.)

## Section S1.

The lower limit of the number of bases for the reading tail mass value was 20. Sequences below the bound were filtered out; meanwhile, a window of 50 bps was set. When the average mass value was lower than 20, the back bases were truncated from the window. The removal of N-containing bases was conducted when the reads below 50 bps were filtered after quality control was complete. The paired reads were merged into a sequence, and the minimum limit of overlap length was 10 bps, based on the overlap relationship between paired-end reads (PE reads).

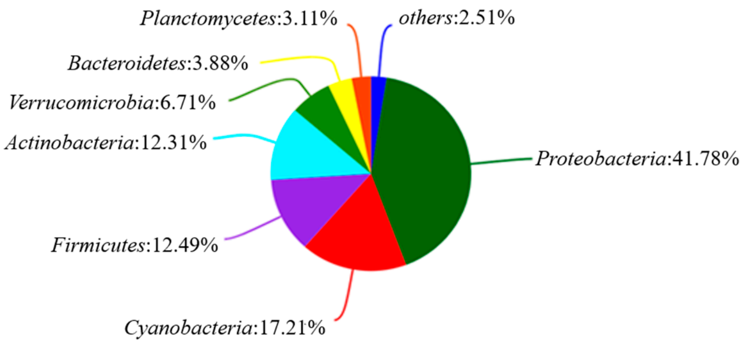


**Figure S1.** Pie pilot of microbial community on phylum level.

(a)

(b)

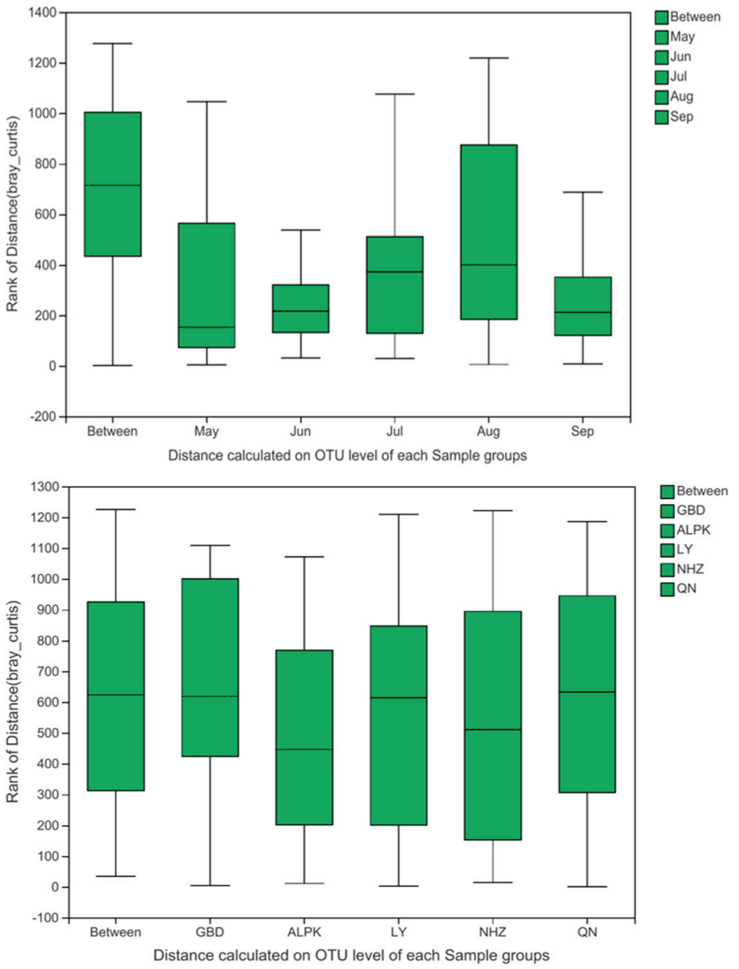


**Figure S2.** Analysis of similarities of microbial community in different groups on OTU level. The corresponding Adonis analysis results exhibited in Table S2.

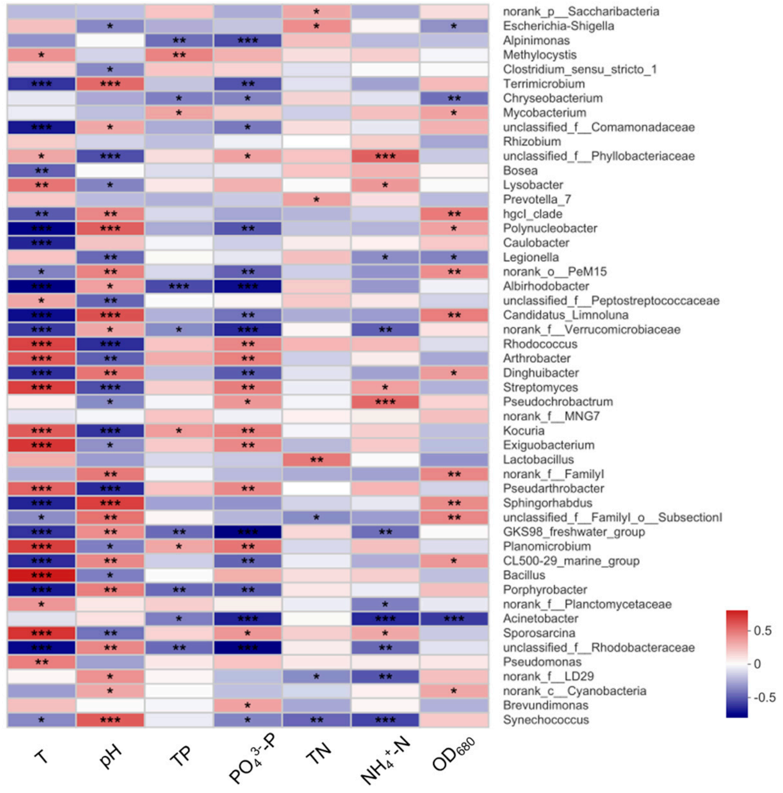

**Figure S3.** Spearman correlation heatmap of primary environmental factors and bacterial community with top 50 bacterial community abundance. $0.01 < p \leq 0.05$, * $0.001 < p \leq 0.01$, *** $p \leq 0.001$.

**Table S1.** TN in different lakes and different layers in five months (unit: mg/L).

| | **May** | **Jun** | **Jul** | **Aug** | **Sep** |
|---|---|---|---|---|---|
| **ALPK** | 1.32±0.03 | 0.52±0.01 | 0.73±0.03 | 0.63±0.02 | 1.76±0.06 |
| **GBD** | 9.75±0.15 | 10.45±0.14 | 11.76±1.24 | 14.52±0.08 | 11.05±0.08 |
| **LY** | 1.06±0.03 | 1.11±0.08 | 1.26±0.04 | 0.99±1.00 | 1.0±0.04 |
| **NHZ** | 1.65±0.01 | 1.48±0.04 | 1.34±0.44 | 1.76±0.06 | 2.03±0.03 |
| **QN** | 5.13±0.10 | 4.40±0.08 | 2.81±0.05 | 2.11±0.06 | 1.05±0.02 |

**Table S2.** Adonis analysis of microbial community in different factor on OTU level

| **Factors** | **Sums Of Sqs** | **Mean Sqs** | **F.Model** | **$R^2$** | **P(>F)** |
|---|---|---|---|---|---|
| Lake | 3.09 | 0.77 | 2.16 | 0.16 | 0.001 |
| Month | 5.22 | 1.31 | 4.19 | 0.27 | 0.001 |
